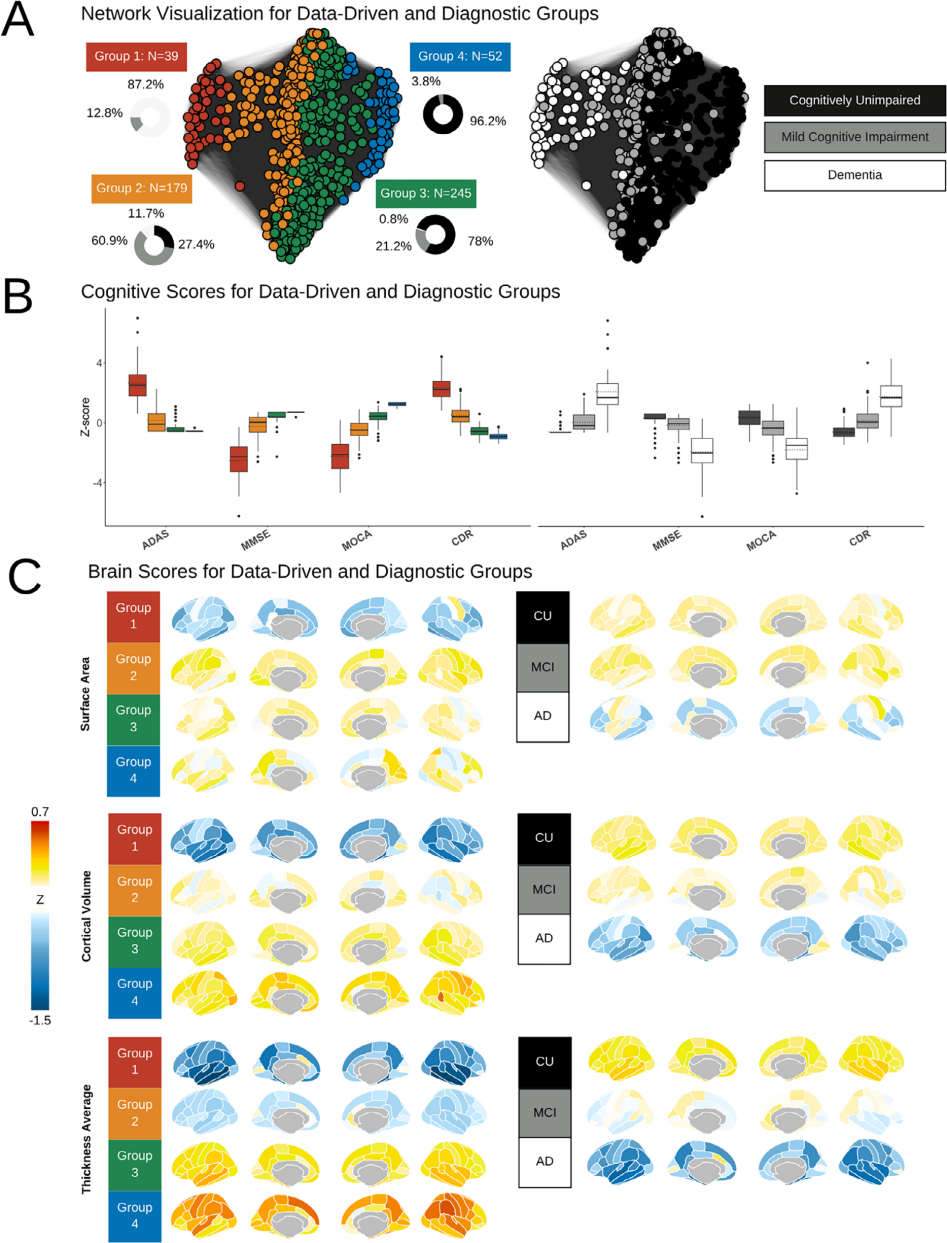# Discovering Hidden Links: Harnessing Similarity Network Fusion to Reveal Common Clusters in Healthy Aging, Mild Cognitive Impairment, and Dementia

**DOI:** 10.1002/alz70856_105149

**Published:** 2026-01-08

**Authors:** Taeko Bourque, Peter Zhukovsky, Cassandra Morrison, John AE Anderson

**Affiliations:** ^1^ Carleton University, Ottawa, ON, Canada; ^2^ CAMH, Toronto, ON, Canada

## Abstract

**Background:**

Cognitively Unimpaired (CU), Mild Cognitive Impairment (MCI), and Alzheimer's Disease (AD) are clinical labels used to categorize degrees of cognitive impairment in the aging brain. Older adults experience brain changes associated with aging and cognitive decline at different ages and progress at varying rates, leading to heterogeneous patterns of cognitive decline. As a result, people within the same diagnostic category often exhibit significant differences in cognitive abilities and brain functions. The goal of the present study was to investigate how data‐driven categories map onto diagnostic categories.

**Method:**

We combined brain imaging features (cortical thickness average, surface area, and volume) with cognitive measures (Alzheimer's Disease Assessment Scale‐Cognitive, Mini‐Mental State Exam, Montreal Cognitive Assessment, Clinical Dementia Rating) in older adults who had a clinical diagnosis of CU, MCI, or AD using Similarity Network Fusion (SNF), a multivariate clustering approach. SNF is a new computational method for data integration that leverages common and complementary information in different types of data. We used data from 515 participants in the Alzheimer's Disease Neuroimaging Initiative (ADNI) database.

**Result:**

We identified four data‐driven groups spanning a gradient of cognitive and neural severity, with an average silhouette width of 0.55, indicating good cluster structure. Group 1 (87% diagnosed with dementia) showed the greatest impairment, while Group 4 (96% cognitively unimpaired) showed minimal impairment. Groups 2 and 3 captured transitional stages, including an “at‐risk” group with early neural and cognitive decline. The top contributing features included the right lingual surface area (NMI = 0.34) and MMSE scores (NMI = 0.052). Significant demographic differences were observed across clusters (e.g., age: F(3, 511) = 30.39, *p* < 0.001).

**Conclusion:**

The current study provides evidence that more nuanced, data‐driven approaches can reveal commonalities in the etiology and underlying neurobiology of individuals across traditional categories of CU, MCI, or AD. These results may lead to more focal therapies and a better understanding of who is at risk for converting to dementia, allowing for earlier detection and treatment of cognitive decline.